# Understanding the molecular–pharmaceutical basis of sartan recalls focusing on valsartan

**DOI:** 10.21542/gcsp.2020.25

**Published:** 2020-11-30

**Authors:** Avik Ray, Shubham Atal, Balakrishnan Sadasivam

**Affiliations:** Department of Pharmacology, All India Institute of Medical Sciences Bhopal, Madhya Pradesh, India

## Abstract

Angiotensin receptor blockers (ARBs) or the ‘sartans’ are widely used for the management of hypertension and heart failure. There have been a series of recent incidents where drug formulations containing different ARBs as active pharmaceutical ingredients have been recalled by various pharmaceutical firms. This article addresses valsartan as well as other sartan recalls besides discussing the recent recalls of ranitidine and metformin, giving insights into the molecular-pharmaceutical basis of the recalls. A thorough literature search of PubMed/Medline and Google Scholar databases was performed to identify all relevant articles and information published up to 29th April 2020 using Medical Subject Headings (MeSH terms) and Boolean operators. We also searched for relevant information on the web using web-browsers and reference lists from original research papers and review articles. The main impurity found was N-nitrosodimethylamine (NDMA) which was thought to be formed due to a change in the manufacturing process of valsartan. Besides, other impurities such N-nitrosodiethylamine (NDEA) and N-nitroso-N-methyl-4-aminobutyric acid (NMBA) were found in batches of other sartans, such as losartan and irbesartan. All of these are carcinogens and harmful if consumed at a level beyond a certain acceptable daily limit. Ranitidine, and more recent metformin recalls, have also been linked with valsartan in view of the presence of NDMA, the same impurity. Safety of ARBs is a major concern among healthcare professionals after the recalls of valsartan in the recent years. Periodic quality assessment of the manufacturing process and the drugs is key to ensure safe, effective and high-quality drugs for the global population. Additionally, practising physicians need to be vigilant in reporting adverse events in their patients receiving treatments.

## Introduction

Valsartan, an angiotensin II receptor antagonist, is primarily used for the treatment of hypertension and heart failure, both as a monotherapy and in combination with other drugs^1,2^. In July 2018, the United States Food and Drug Administration (USFDA) alerted the world regarding the voluntary recalls of many drug formulations containing valsartan as an active ingredient. The recalls were due to the presence of an impurity known as N-nitrosodimethylamine (NDMA), a recognized probable human carcinogen^3^. However, not all products containing valsartan were recalled; the contamination was specific for formulations manufactured by a Chinese drug manufacturer, Zhejiang Huahai Pharmaceuticals (ZHP). It seemed to be associated with a change in the steps of the manufacturing process of the company which were implemented in 2012. The impurity was found incidentally while performing other tests and not through routine assessment performed during batch release. As a result, the European Medicines Agency (EMA) along with the USFDA withdrew all the concerned product batches from the market^3,4^.

### Literature search and results

Literature search of PubMed and Google Scholar databases was performed for articles and information as available up to 19th April 2020. It was carried out by using combinations of the following keywords: ‘valsartan’, ‘sartan’, ‘impurities’, ‘recall’, ‘drug’ and ‘FDA’. While searching in PubMed, Medical Subject Headings (MeSH terms), subheadings and “All Fields” were combined with key Boolean operators “AND”, “OR” and “NOT” to get the relevant studies and exclude the studies which did not match our requirements. However, since we did not conduct a systematic review, there was no stringent inclusion and exclusion criteria.

Nevertheless, articles were selected only if they imparted information on valsartan recalls and impurities irrespective of their type. The relevance of the articles for their inclusion was assessed and reviewed independently by two authors (AR and SA). Recent newspaper articles and press releases as obtained from Google were also searched for relevant information and were included only if they were related to valsartan recalls and impurities. Further, reference lists from the original research papers and review articles were checked for relevance. Data was synthesized from the sources and reviewed by at least two authors for inclusion in different sections of this review.

A total of one hundred and sixty-one (161) publications and relevant articles were obtained from the electronic databases and web pages searched. After removal of the duplicates, eighty-six (86) were considered for further screening and evaluation. The title and abstracts of the publications were carefully assessed. Thirty-five (35) publications and articles were further eliminated since they were not completely relevant to the topic. Finally, fifty-one (51) published articles and recent newspaper reports and press releases as obtained from Google were considered for this review. Amongst the included published studies, publication date ranges from October 2018^5^ to February 2020^6^.

### Therapeutic profile of valsartan as a prototype angiotensin receptor blocker (ARB)

The main indications for the administration of valsartan are hypertension, both adult and pediatric, heart failure, and post-myocardial infarction. It was originally developed by Novartis International AG and was sold under the brand name of DIOVAN. It was labelled as the world’s most selling anti-hypertensive medication and resulted in a $6 billion in sales in 2010 worldwide^7^. The patent for both valsartan/hydrochlorothiazide expired in September 2012, making it a prime target for the generic industries^8^.

Valsartan is a potent and orally active non-peptide tetrazole derivative. It is a selective angiotensin II receptor antagonist which is lipophilic in nature and has an intermediate onset of action as compared to other drugs of the same category. It belongs to class III of the Biopharmaceutics Classification System (BCS) - as a low permeability and high lipid solubility drug. It is rapidly absorbed after oral administration, has a low volume of distribution and is extensively bound to the plasma proteins. It is mainly eliminated by non-renal routes. When administered orally, valsartan is mainly eliminated in faeces (about 83% of the dose) and urine (about 13% of the dose). The elimination is mostly as unchanged form, with only 20% of the dose recovered as metabolite. The primary metabolite, valeryl-4-hydroxy valsartan accounts for only 9% of the dose^9^.

Valsartan monotherapy with 80 mg as the initial dose has shown considerable effectiveness in hypertensive patients, particularly those with congestive heart failure (CHF) and renal impairment, and as an alternative and/or add-on therapy in hypertensive patients not responding to or tolerating ACE inhibitors and β-blockers or diuretics, respectively^10^. The therapeutic focus in managing hypertensive patients now includes end-organ protection as an equally important treatment goal as reduction of blood pressure (BP) which is provided by valsartan.

Valsartan has beneficial effects in heart failure by blocking the angiotensin type 1 (AT_1_) receptors. There is down-regulation and reduced gene expression of the AT_1_ receptors in heart failure which leads to enhanced local activity of angiotensin II^11^. Valsartan therapy with initial dose of 40 mg twice daily oral dose has shown significantly reduced hospitalization rates for heart failure^12^.

### Valsartan synthesis and purity

Valsartan has been listed in different pharmacopoeias worldwide along with the methods to test identity, purity and perform assay. Usually, purity testing focuses mainly on known or expected impurities from synthesis and/or degradation. Currently, the purity testing for valsartan focuses on enantiomeric purity, the concerned compounds being ent-Valsartan [European Pharmacopoeia (Ph. Eur.), impurity A], valsartan benzyl ester (Ph. Eur., impurity B), and desmethylvalsartan (the butyl analogue of valsartan, Ph. Eur. impurity C). Both the United States Pharmacopoeia (USP) and Ph.Eur. consider residual water too as an impurity in the manufacturing process of valsartan, while sulphated ash has been proposed to be considered an impurity by Ph.Eur^13,14^.

**Figure 1.**
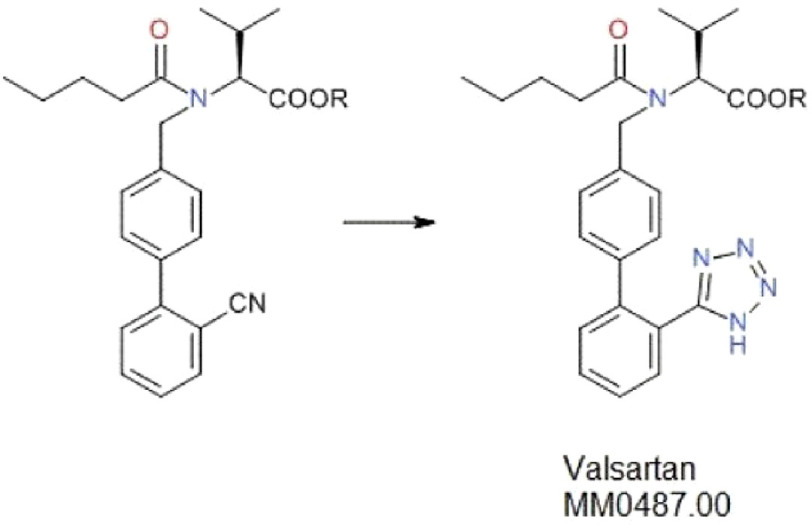
Generalized diagram to show the formation of the tetrazole moiety of valsartan. Tetrazole is built up at the very end of the synthesis process of valsartan. This makes it more likely that an impurity formed within this step is still present in the final product. Usually azides are involved in this reaction e.g., sodium azide in dimethylformamide (DMF) at relatively high temperatures. MM0487.00 is the Stock Keeping Unit (SKU) of valsartan used as a reference for the pure form of the compound and the CAS (Chemical Abstracts Service) registry number is 137862-53-4.

The synthesis of valsartan (Figure 1) begins from (S)-valin methyl ester and 4’-(bromomethyl)-[1,1’-biphenyl]-2-carbonitrile or 2-cyano-4-formylbiphenyl as mentioned in the Ph. Eur. commentary^13^. The tetrazole moiety is formed in the final step by reacting with azidotributyltin (IV)^15^. Several other routes for the synthesis of valsartan have been published over the past few years^16–28^. They represent two different principles for synthesis; formation of tetrazole ring from a cyano intermediate which is analogous to the route described by the Ph. Eur., and biphenyl coupling by using an activated tetrazole as educt. Multiple patent applications for synthesis processes have been submitted. A few mention the use of sodium nitrite (NaNO2) in product generation and removal of azide^29–31^. The patent application of ZHP, the Chinese manufacturer in question, claims the formation of tetrazole using anhydrous zinc chloride and sodium azide (NaN3), preferably in dimethylformamide (DMF) as a solvent, followed by quenching with NaNO_2_^32^. DMF has limited stability and this might have resulted in traces of dimethylamine followed by the formation of NDMA^33^. Therefore, Certification of Suitability (CEP) for this particular method seems to be inappropriate. The use of NaN_3_ leads to higher yield of valsartan as compared to the original patented method followed by Novartis using tributyltin azide^34^ which might have been the reason for ZHP to adopt this method.

An analysis of various valsartan containing drug formulations in Germany was performed and an NDMA concentration of up to 22 µg per tablet, corresponding to ∼60 mg/kg of valsartan Active Pharmaceutical Ingredient (API), was found^35^. The USFDA reported, through an official update titled “Analysis of N-nitrosodimethylamine (NDMA) Levels in Recalled Valsartan in the U.S.” in July 2018, that the concentration of NDMA impurities was considerably higher in batches of valsartan in which the API was supplied by the ZHP as compared to other manufacturing companies^36^. Generally, N-nitrosoamines (NAs) are products of reaction of nitrites with amines, usually generated at elevated temperature. Hence, they are generally detected in foods and drinks after processing. NAs also appear in ground and drinking water as a by-product of disinfection, with a cut-off level in the lower range. The World Health Organization (WHO) has set the maximum level of NDMA in drinking water at 100 ng/L^37^.

### N-nitrosodimethylamine (NDMA): Points of note

**Figure 2.**
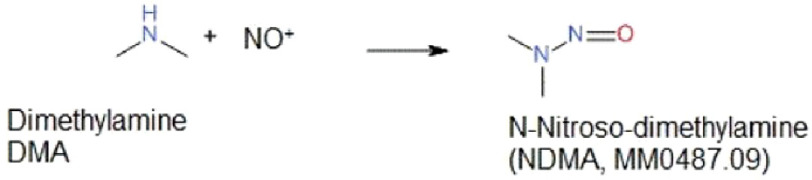
The synthesis of NDMA impurity. One potential source of NDMA could be the degradation of dimethylformamide (DMF) under harsh conditions and reaction with sodium nitrite NaNO_2_. In such a reaction dimethylamine (DMA) would react with a nitrosonium ion to form NDMA. MM0487.09 and 62-75-9 are the Stock Keeping Unit (SKU) and CAS (Chemical Abstracts Service) registry number of NDMA respectively.

N**-**Nitrosodimethylamine (NDMA), which is also known as dimethylnitrosamine (DMN), is a semi-volatile organic compound (Figure 2). The WHO International Agency for Research on Cancer (IARC) and the U.S. Environmental Protection Agency (EPA) have classified it as a probable carcinogen to humans and a toxic agent^38–43^. It is used to induce cancer in rats for various anti-cancer drug screening studies^44^. NDMA is suspected to have both systemic as well as localized carcinogenic effects due to its property of inducing deoxyribonucleic acid (DNA)-damaging radicals in the gastrointestinal tract and liver^45,46^. It is metabolized in the liver by cytochrome P (CYP) 450 enzyme CYP2E1 to methyldiazonium that leads to mutations by acting as a methylating agent^47^. N-nitroso compounds also lead to the activation of ras oncogenes which are thought to be associated with the development of colon cancer^45^. Carcinogenic dose in rats was found out to be around 10 µ g/kg/day^47^. No human experiments have been performed to date; however, it is known that some food products such as processed meat do contain small amount of NDMA. Based on this knowledge, various studies based on food frequency questionnaires have been performed. In three such studies, increased risk of development of colorectal carcinoma was found to be associated with NDMA exposure^48–50^. A Danish nationwide cohort study was performed taking all cancers except non-melanoma skin cancer as the primary composite endpoint and the exposure to NDMA as the risk factor under assessment. The results showed increased risk for single cancer outcomes, namely, colorectal cancer and uterine cancer^51^.

With regards to the valsartan recalls, the USFDA stated that “some levels of the impurity may have been in the valsartan-containing products for as long as four years. USFDA scientists estimate that if 8000 people took the highest valsartan dose (320 mg) from the recalled batches daily for the full 4 years, there may be one additional case of cancer over the lifetimes of these 8000 people”^52^. This impurity is not only restricted to sartan molecules. Ranitidine shows the highest molar conversion and formation of NDMA^53^.

The other manufacturers apart from the Chinese Manufacturer ZHP who were found to have produced the impure sartans are Hetero Labs, Mylan Laboratories, and Aurobindo Pharma. Notably, all of these manufacturers are based in India. Though each of them produced valsartan following different methods, they all had NDMA as an impurity in the active pharmaceutical ingredient^34^. According to the USFDA, Hetero Labs uses a manufacturing process for synthesizing valsartan quite similar to ZHP’s but the levels of NDMA detected in their products have been generally much lower than those found in the ZHP products. However, the USFDA did not confirm the reason for the formation of this impurity but stated that an investigation is ongoing^34^.

On 24th September 2018, the USFDA released a gas chromatography-mass spectrometry (GC/MS) headspace method for pharmaceutical manufacturers to detect NDMA and quantify its amount in valsartan API and other suspected drug products^54^.

### The recalls

Table 1 shows the timeline of various drugs recalled by different pharmaceutical companies, as uploaded on the official website of the USFDA^55^. In December 2018 and January 2019, multiple batches of valsartan tablets of two reputed Indian drug manufacturers were recalled citing ‘cancer concerns’. Aurobindo Pharma USA, Inc. had recalled 80 lots of valsartan on 31st of December, 2018 while Torrent Pharmaceuticals Limited expanded its voluntary recall from 2 lots of losartan potassium tablets USP to a total of 10 lots as on the 3rd of January, 2019^56,57^.

**Table 1:** The timeline of recalls of ‘sartan’ containing drug formulations.

**Date**	**Brand (if mentioned),**	**Formulation**	**Reason for**	**Type (voluntary/**
	**Company**		**recall**	**involuntary)**
**07/16/2018**	Major Pharmaceuticals	Valsartan tablets, 80 mg	May contain the probable	Voluntary
		USP and 160 mg USP	carcinogen NDMA	
**07/18/2018**	Prinston	Valsartan Tablets, 40	Detection of a trace	Voluntary
	Pharmaceutical Inc. dba	mg, 80 mg, 160 mg, and	amount of unexpected	
	Solco Healthcare LLC	320 mg; and Valsartan-	impurity, NDMA	
		Hydrochlorothiazide		
		Tablets, 80 mg/12.5 mg,		
		160 mg/12.5 mg,		
		160 mg/25 mg,		
		320 mg/12.5 mg, and		
		320 mg/25 mg		
**11/09/2018**	Sandoz Inc.	Losartan Potassium	Due to detection of	Voluntary
		Hydrochloride	NDEA impurity	
**11/27/2018**	Teva Pharmaceuticals	Amlodipine/Valsartan	Due to detection of	Voluntary
		Combination Tables	NDEA impurity	
		and Amlodipine		
		/Valsartan/Hydrochloro		
		thiazide Combination		
		Tablets		
**12/04/2018**	Mylan Pharmaceuticals	Valsartan-containing	Due to detection of	Voluntary
		products	NDEA impurity	
**12/20/2018**	Torrent	Losartan potassium	Due to detection of	Voluntary
	Pharmaceuticals	tablets, USP	NDEA impurity	
	Limited			
**12/31/2018**	Aurobindo Pharma	Amlodipine Valsartan	Due to detection of	Voluntary
	USA, Inc.	Tablets USP, Valsartan	NDEA impurity	
		HCTZ Tablets USP,		
		Valsartan Tablets USP		
**01/03/2019**	Torrent	Losartan potassium	Detection of trace	Voluntary
	Pharmaceuticals	tablets, USP	amounts of NDEA	
	Limited			
**01/18/2019**	Prinston	Irbesartan and	Due to detection of	Voluntary
	Pharmaceutical Inc. dba	Irbesartan HCTZ tablets	NDEA impurity	
	Solco Healthcare LLC			
**01/22/2019**	Torrent	Losartan potassium	Lack of Sterility	Voluntary
	Pharmaceuticals	tablets, USP; Losartan		
	Limited	potassium		
		hydrochlorothiazide		
		tablets, USP		
**02/22/2019**	Macleods	Losartan	Due to detection of	Voluntary
	Pharmaceuticals	Potassium/Hydrochloro	NDEA impurity	
	Limited	thiazide Combination		
		Tablets		
**02/28/2019**	Camber	Losartan tablets, USP;	Due to detection of	Voluntary
	Pharmaceuticals, Inc.	25 mg, 50 mg and 100	NMBA impurity	
		mg		
**03/01/2019**	Aurobindo Pharma	Valsartan and	Due to detection of	Voluntary
	USA, Inc.	Amlodipine tablets;	NDEA impurity	
		Valsartan Tablets		
**03/01/2019**	Torrent	Losartan potassium	Due to detection of	Voluntary
	Pharmaceuticals	tablets, USP and	NMBA impurity	
	Limited	Losartan potassium		
		hydrochlorothiazide		
		tablets, USP		
**03/01/2019**	Acetris, Aurobindo	Valsartan and	Due to the detection of	Voluntary
	Pharma USA, Inc.	Amlodipine and	NDEA impurity	
		Valsartan tablets		
**03/07/2019**	Aurobindo Pharma	Valsartan Tablets USP	Due to the detection of	Voluntary
	USA, Inc.		NDEA	
**03/19/2019**	Legacy, Legacy	Losartan potassium	Contain NMBA	Voluntary
	Pharmaceutical	tablets, USP		
	Packaging			
**03/28/2019**	Legacy, Legacy	Losartan potassium	Contain NMBA	Voluntary
	Pharmaceutical	tablets, USP		
	Packaging			
**04/18/2019**	Torrent	Losartan potassium	Due to detection of	Voluntary
	Pharmaceuticals	tablets, USP; Losartan	NMBA impurity	
	Limited	potassium and		
		Hydrochlorothiazide		
		tablets, USP		
**04/26/2019**	GSMS Incorporated,	Losartan Potassium 25	Due to detection of	Voluntary
	Teva Pharmaceuticals	mg and 100 mg Tablets	NMBA impurity	
	USA, Inc.	USP		
**05/03/2019**	Heritage, Vivimed	Losartan Potassium	Due to detection of	Voluntary
		Tablets USP	NMBA impurity	
**06/11/2019**	GSMS Incorporated,	Losartan potassium	Due to detection of	Voluntary
	Teva Pharmaceuticals	tablets	NMBA impurity	
	USA, Inc.			
**06/26/2019**	Macleods	Losartan Potassium	Due to detection of	Voluntary
	Pharmaceuticals	USP tablets and	NMBA impurity	
	Limited	Losartan		
		Potassium/Hydrochloro		
		thiazide combination		
		tablets		
**07/15/2019**	Legacy Pharmaceutical	Losartan Potassium	Due to detection of	Voluntary
	Packaging	USP	NMBA impurity	
**09/19/2019**	Torrent	Losartan Potassium	Due to detection of	Voluntary
	Pharmaceuticals	Tablets, USP and	NMBA impurity	
	Limited	Losartan		
		Potassium/Hydrochloro		
		thiazide Tablets, USP		
**03/17/2020**	Aurobindo Pharma	Irbesartan	Due to detection of	Voluntary
	USA, Inc.		NDEA impurity	
**03/19/2020**	Torrent	Valsartan/Amlodipine/	Impurity (not specifically	Voluntary
	Pharmaceuticals	HCTZ;	mentioned)	
	Limited	Valsartan/Amlodipine;		
		and Valsartan tablets		
**03/20/2020**	Torrent	Valsartan/Amlodipine/	Due to detection of	Voluntary
	Pharmaceuticals	HCTZ Tablets	NMBA impurity	
	Limited			
**03/20/2020**	Camber	Valsartan Tablets, USP,	Detection of Trace	Voluntary
	Pharmaceuticals, Inc.	40 mg, 80 mg, 160 mg	Amounts NDMA	
		and 320 mg	Impurity	
**03/24/2020**	Actavis, Teva	Valsartan and Valsartan	Impurity (not specifically	Voluntary
	Pharmaceuticals USA	Hydrochlorothiazide	mentioned)	
		Tablets		

**Notes.**

USP United States Pharmacopeia NDMA N-nitrosodimethylamine LLC Limited liability company HCTZ Hydrochlorothiazide NDEA N-nitrosodiethylamine API Active Pharmaceutical Ingredient NMBA N-Nitroso-N-Methyl-4 amino butyric acid

Legacy Pharmaceutical Packaging, LLC had recalled 40 repackaged lots of losartan tablets, USP due to the detection of trace amounts of N-Nitroso N-Methyl 4-amino butyric acid (NMBA), another possible process impurity or contaminant in the API^58^. DMF has also been identified in valsartan tablets manufactured by several companies including Novartis International AG and has been found in valsartan formulations which are still in the U.S. market, including the medicines that the USFDA had highlighted as alternatives to the recalled batches of valsartan. DMF is classified by the WHO as a probable carcinogen. Hence this finding can complicate USFDA’s efforts to pull out the tainted drugs from the market while informing the physicians and the patients which medications are safe^59^.

Likewise, various drugs belonging to the ‘sartan’ group were recalled in Europe as well based on the upper limit set for NDMA and NDEA concentration (Table 2). EMA and other national authorities in various European countries are currently investigating ways to detect the presence of nitrosamine impurities in drugs, including other related impurities such as N-nitrosoethylisopropylamine (EIPNA) and N-nitrosodiisopropylamine (DIPNA). The European authorities will also consider the lesson learnt from these recalls and reviews to improve the methods of identifying and handling impurities in drugs^60^. As of April 2019, a total of 249 active pharmaceutical ingredient batches and 2000 medicinal product batches have been tested for NDMA impurity while 637 active pharmaceutical ingredient batches and 1007 medicinal product batches have been tested for NDEA. The levels of nitrosamine impurities in quite a number of products came out to be out-of-specification (Table 3)^61^.

**Table 2:** The acceptable limits of NDMA and NDEA impurities.

	**NDMA**		**NDEA**	
**Active Substance**	**Maximum**	**Limit**	**Maximum**	** Limit (ppm)**
**(Max Daily Dose)**	**daily intake**	**(ppm)**	**daily intake**	
	**(ng)**		**(ng)**	
Candesartan (32 mg)	96.0	3.000	26.5	0.820
Irbesartan (300 mg)	96.0	0.320	26.5	0.088
Losartan (150 mg)	96.0	0.640	26.5	0.177
Olmesartan (40 mg)	96.0	2.400	26.5	0.663
Valsartan (320 mg)	96.0	0.300	26.5	0.082

**Notes.**

NDMA N-nitrosodimethylamine NDEA N-nitrosodiethylamine ppm parts per million ng nanograms

**Table 3:** Number of out-of-specification products based on the levels of nitrosamine impurities.

	Valsartan		Losartan		Irbesartan	
** Impurity**	**API**	**Medicinal product**	**API**	**Medicinal product**	**API**	**Medicinal product**
**NDMA**	70 out of 141	253 out of 621	0 out of 16	0 out of 312	0 out of 20	0 out of 260
**NDEA**	53 out of 200	36 out of 246	1 out of 149	2 out of 188	28 out of 160	29 out of 175
**NMBA**	–	–	13 out of 72	–	–	–

**Notes.**

NDMA N-nitrosodimethylamine NDEA N-nitrosodiethylamine NMBA N-Nitroso-N-Methyl-4 amino butyric acid API Active Pharmaceutical Ingredient

### Ranitidine recalls and its link with valsartan

On September 13th 2019, the USFDA released an official statement to alert the patients and the healthcare professionals about NDMA found in a few samples of one of most commonly prescribed over-the-counter medicine used to decrease gastric acid production, ranitidine. This was followed by a voluntary recall of 14 lots of ranitidine capsules (Zantac) distributed by Sandoz Inc. Notably, the ‘sartan’ recalls, which were due to presence of the same impurity, were slowly dying down by this time. In the coming months, multiple recalls by various pharmaceutical firms took place while alternative medications such as famotidine and cimetidine came out to be free of the impurity. Later on, another alternative drug called nizatidine came under the radar and the USFDA further alerted regarding voluntary recall of nizatidine capsules distributed by Mylan. Finally, on 1st April 2020, the USFDA requested the removal of all ranitidine products from the US market in view of NDMA concentration increasing over time, especially when stored at higher than room temperature, thus exposing the consumers to unacceptable levels of the impurity^62^. This goes onto to show how periodic quality and safety testing by the manufacturing firms are important in identifying a potential carcinogenic impurity in the medicines produced. This calls for attention from the global regulatory bodies to formulate more stringent pharmaceutical policies^63^. Recently, a few pharmaceutical firms have recalled back batches of extended-release formulations of metformin due to the presence of the same impurity beyond acceptable limits^64^, highlighting the importance of stringent monitoring of NDMA levels in all probable drugs henceforth.

### Other related impurities found in the ‘sartans’

As mentioned earlier, valsartan is a tetrazole motif^15^. There are several methods by which production of hazardous by-products can be prevented. Cleaning with chloramine is a common method adopted to reduce the formation of disinfection by-products (DBPs) such as trihalomethanes (THMs) and haloacetic acids (HAAs)^46^. Another impurity, namely N-nitrosodiethylamine (NDEA), was detected in losartan API manufactured by Hetero Labs^65^.

**Figure 3.**
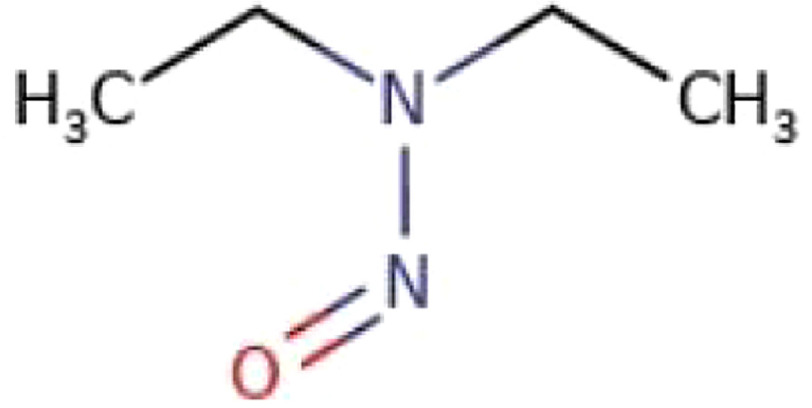
Structure of N-nitrosodiethylamine (NDEA). The CAS (Chemical Abstracts Service) registry number is 55-18-5.

Analyses of these impurities in four other ARBs, namely Candesartan, Irbesartan, Losartan and Olmesartan have been undertaken in Germany^66^. Like NDMA, NDEA is also considered a carcinogen for humans (Figure 3). The Committee for Medicinal Products for Human Use (CHMP) currently considers a daily intake of 96 ng/day for NDMA and 26.5 ng/day for NDEA as maximum intake levels which corresponds to 0.3 ppm for NDMA and 0.08 ppm for NDEA in valsartan 320 mg tablets^66^. The NDMA levels in the recalled tablets ranged from 0.3 to 16 ppm per valsartan 320 mg tablets, clearly highlighting the reason for their recalls^67^.

## Limitations

There are certain limitations to this review. The exact metrics or figures in some cases could not be mentioned due to non-availability of accurate data even after thorough literature search. The chemical synthesis pathways of valsartan or other sartans have not been described in detail; the relevant steps have been discussed in brief. The exact economic burden due to drug recalls, which might have given an additional insight regarding the importance of the post-marketing surveillance and monitoring in such situations, could not be included in the discussion as the required data was not available.

## Conclusions

The practice of changing the chemical processes to synthesize the same API is very common because of flexible and non-rigid pharmaceutical process patenting policies^68^. As evident from the valsartan recall (the key points have been summarized in Table 4), this practice can lead to formation of impurities in other drugs as well. We can expect more drugs to be detected with impurities and recalled in the near future unless more stringent policies and regulations are introduced in the process patenting procedures. Recall of drugs used for the management of common conditions such as hypertension is a very serious issue. Drug impurities which are being detected are serious enough to warrant these recalls, but halting the use of a drug without medical supervision can cause other serious medical problems for the patients. Along with strict regulatory oversight, drug manufacturers, physicians and pharmacists also need to play an important role in detecting and reporting potential adverse drug events associated with any drug formulation. This can help local and international drug safety and regulatory bodies to act and take appropriate measures. The ‘sartan’ recalls once again remind us that safety of the consumers is of utmost importance and vigilance is required at all levels, starting from manufacturing till the consumption of drugs. Besides, the recalls of ranitidine and metformin, two of the commonly prescribed medicines given to patients suffering from various cardiovascular ailments to treat heartburn and diabetes mellitus respectively, emphasize the importance of dissipating the knowledge to the healthcare professionals across the world and educate them to be vigilant in reporting any suspected adverse event of their patients receiving treatments.

**Table 4:** An overview of the important findings.

S. No.	Key Points
1.	Starting from July 2018, there have been numerous voluntary recalls of batches of valsartan containing drug formulations.
2.	These recalls were due to the unwanted presence of carcinogenic nitrosoamine impurities - N-Nitrosodimethylamine (NDMA), N-nitrosodiethylamine (NDEA) and N-Nitroso N-Methyl 4-amino butyric acid (NMBA).
3.	The recalls later extended to other ‘sartans’ as well—losartan and irbesartan
4.	This resulted in the USFDA releasing a gas chromatography-mass spectrometry (GC/MS) headspace method for pharmaceutical manufacturers to detect and quantify NDMA impurities in valsartan containing products and other drug formulations in September 2018.
5.	Recently, detection of NDMA impurities have led to voluntary recalls of ranitidine capsules (Zantac) distributed by Sandoz Inc. and extended-release formulations of metformin.
6.	Drug recalls are important nuisances which should be dealt with cautiously. Post-marketing surveillance should closely monitor for any impurity related drug issues for prompt reporting. Healthcare professionals across the world should be vigilant in reporting any suspected adverse event of their patients receiving treatments.

## Author contributions

AR, SA and BS contributed equally to conception, analysis, and composition.

## Declaration of conflicting interests

The Authors declares that there is no conflict of interest.

## Funding

This research received no specific grant from any funding agency in the public, commercial, or not-for-profit sectors.
